# Developing Individualized Follow‐Up Strategies Based on High‐Risk Recurrence Factors and Dynamic Risk Assessment for Locally Advanced Rectal Cancer

**DOI:** 10.1002/cam4.70323

**Published:** 2024-10-28

**Authors:** Jianjian Qiu, Yilin Yu, Zhiping Wang, Liang Hong, Lingdong Shao, Junxin Wu

**Affiliations:** ^1^ Department of Radiation Oncology Clinical Oncology School of Fujian Medical University, Fujian Cancer Hospital Fuzhou China

**Keywords:** conditional survival, dynamic risk assessment, high‐risk recurrence factors, individualized follow‐up strategies, locally advanced rectal cancer

## Abstract

**Background:**

Locally advanced rectal cancer (LARC) is one of the most common malignant tumors worldwide, and its incidence is increasing year by year. Despite multimodal treatment, the recurrence rate of LARC patients remains high, about 20%–50%. However, the follow‐up strategy according to tumor stage has certain limitations. There is no consensus on the optimal frequency and duration of follow‐up. This study aims to comprehensively analyze the high‐risk factors for recurrence in LARC from clinical characteristics, nutritional indicators, and imaging indexes. It intends to utilize conditional survival (CS) evaluation to assess dynamic survival and recurrence risks after comprehensive treatment of LARC and to develop individualized follow‐up strategies.

**Methods:**

Logistic regression was utilized to analyze the independent recurrence factors in LARC patients. Calibration curve, decision curve, and ROC curve were employed to evaluate the model's efficacy. Kaplan–Meier curve was used to calculate CS rate and compare survival differences among different risk groups.

**Results:**

A total of 561 patients were analyzed in our study. Our multivariable logistic regression analysis revealed that the prognostic nutritional index (PNI), extramural vascular invasion (EMVI), vascular tumor thrombus, perineural invasion, and tumor size were independent factors for recurrence. Subsequently, a nomogram model was constructed and risk stratification was performed. Calibration curves and decision curves demonstrated that the model exhibited good clinical efficacy. The area under the ROC curve for the model was 0.763, indicating good sensitivity and specificity. Kaplan–Meier curves showed significant differences in survival among different risk groups. Furthermore, we observed that the CS without local recurrence and distant metastasis increased each year, while the cumulative recurrence risk decreased annually with prolonged survival time. Tailored follow‐up intensities were developed for different risk groups and clinical stages based on the cumulative recurrence risk.

**Conclusion:**

The personalized follow‐up strategy based on risk stratification can optimize resource allocation, early detection of recurrence or metastasis, and ultimately enhance the overall care and prognosis of LARC patients.

**Abbreviations**
AJCC: American Joint Committee on Cancer
BMI: body mass index
CI: confidence interval.
CS: conditional survival
DCA: decision curve analysis
DMFS: distant metastasis‐free survival
EMVI: extramural vascular invasion
LARC: local advanced rectal cancer
LRRFS: local‐regional recurrence‐free survival
MRF: mesorectal fascia invasion
OR: odds ratio
OS: overall survival
PNI: prognostic nutritional index
RC: rectal cancer
ROC: receiver operating characteristic curve
UICC: Union for International Cancer Control

## 1 Introduction

Colorectal cancer is one of the most common malignancies globally, with its incidence steadily increasing [1]. Among them, rectal cancer (RC) patients accounted for a significant proportion and exhibited a trend toward affecting younger individuals [2]. Local advanced rectal cancer (LARC) poses significant challenges in terms of treatment and prognosis. While surgery remains the cornerstone of colorectal cancer treatment, especially in early stages, the management of LARC necessitates a multimodal approach to achieve optimal outcomes [3]. Over the past few decades, the advent of neoadjuvant therapies such as radiotherapy, chemotherapy, and targeted treatments have revolutionized the treatment landscape for LARC, significantly improving patient outcomes [4, 5, 6]. Neoadjuvant therapies play a crucial role in tumor downstaging, increasing the likelihood of complete resection, and reducing the risks of local recurrence and distant metastasis [7, 8]. However, despite significant advancements in neoadjuvant therapies, patients with LARC still face a high risk of disease recurrence (20%–50%) and distant metastasis (25%–30%) postoperatively [9, 10]. Therefore, the follow‐up strategy after comprehensive treatment of LARC is crucial for the early detection of recurrence or metastasis and timely intervention to improve the prognosis and quality of life of patients.

Currently, there is a wide variety of posttreatment follow‐up strategies for patients with LARC after neoadjuvant therapy. Follow‐up typically includes routine imaging studies, monitoring of tumor markers, and clinical assessments. However, there is a lack of consensus regarding the optimal frequency and timing of follow‐up, as well as the most appropriate imaging modalities and biomarkers to effectively monitor disease progression. Imaging examination (such as CT and MRI), tumor marker monitoring (such as CEA and CA19‐9), and clinical evaluation (such as physical examination and symptom evaluation) are commonly used in follow‐up. Follow‐up visits at 3 months after comprehensive treatment are currently recommended as a baseline, every 3 months for 2 years, every 6 months for 3–5 years, and then annually. Furthermore, the follow‐up models recommended in clinical guidelines have not yet taken into account differences between risk groups [11, 12]. Nevertheless, there is a scarcity of research on comprehensive posttreatment follow‐up strategies for patients with LARC. Conditional survival (CS) is a novel survival predictor, which can dynamically assess the risk of recurrence and death after treatment. It is defined as the probability of surviving for several years after the patient has survived for a specified time. For example, the 3‐year CS probability of a patient surviving year 1 is the likelihood that the patient will survive the next 3 years given that he or she has survived for 1 year. By analyzing the survival probability of patients in a specific time period, it provides important practical value and potential for clinical practice.

At present, there are still insufficient studies on CS‐based personalized follow‐up strategies for LARC patients after comprehensive treatment. Therefore, this study aims to analyze the high‐risk factors of recurrence in LARC patients from the aspects of clinical characteristics, nutritional indicators, and imaging indicators and to evaluate the recurrence risk and CS of patients in different risk groups based on CS, so as to develop personalized follow‐up strategies.

## 2 Materials and Methods

### 2.1 Patients

We retrospectively collected data on patients with LARC who underwent surgery after receiving neoadjuvant therapy at our hospital between 2014 and 2020. Inclusion criteria were as follows: (1) pathologically confirmed RC; (2) postoperative Stage II or III; (3) age ≥ 18 years; (4) no history of other malignancies. Exclusion criteria were as follows: (1) patients with severe infections or other major illnesses before treatment (e.g., liver or kidney failure, severe cardiovascular or cerebrovascular diseases); (2) incomplete clinical data; (3) loss to follow‐up. Staging was performed for all patients according to the eighth edition of the American Joint Committee on Cancer (AJCC)/Union for International Cancer Control (UICC) TNM staging system. Clinical T and N staging for all patients were determined through a comprehensive analysis of all examination results by at least two experienced clinicians. Ultimately, 561 patients met the inclusion criteria. This study was conducted in accordance with the principles of the Declaration of Helsinki and was approved by the Ethics Committee of the Fujian Cancer Hospital.

### 2.2 Data Collection

We conducted a retrospective analysis using clinical data from our institution. Data obtained from the hospital's electronic systems included clinicopathological information as well as imaging data. The clinicopathological data comprised patient demographics (age, gender), postoperative staging, tumor markers, body mass index (BMI), prognostic nutritional index (PNI), while imaging data included details on extramural vascular invasion (EMVI), mesorectal fascia invasion (MRF), perineural invasion, vascular tumor thrombus, and tumor deposits. Blood routine and blood biochemistry were collected before treatment, and the nutritional status of patients before treatment was evaluated according to the levels of lymphocytes and albumin. The formulas for calculating BMI and PNI, two nutrition‐related indicators, are as follows: BMI, patient's weight (kilograms)/the square of their height (meters); PNI, 5 × lymphocyte count + serum albumin.

### 2.3 Study Endpoints and Follow‐Up

The primary endpoints of this study are the local–regional recurrence‐free survival (LRRFS) and distant metastasis‐free survival (DMFS), with overall survival (OS) as a secondary endpoint. LRRFS was defined as the time from diagnosis to occurrence of local or regional recurrence, death, or last follow‐up, whereas DMFS was defined as the time from diagnosis to first occurrence of distant metastasis, death, or last follow‐up, whichever occurred first. OS is defined as the time from diagnosis to death from any cause or the end of the last follow‐up.

### 2.4 Follow‐Up Strategy

In general, the follow‐up schedule for RC patient posttreatment typically involves the following: within the first year after treatment, follow‐up appointments are usually scheduled every 3 months; in the second and third years, the frequency reduces to every 6 months; and in subsequent years, it becomes once a year. During the follow‐up process, the following examinations are typically conducted: physical examination, blood tests (biochemistry, complete blood count, tumor markers), imaging tests (CT scan, MRI, or PET‐CT), and, if necessary, endoscopic examinations. The last follow‐up for this study was in June 2023.

### 2.5 Conditional Survival

CS refers to the probability of surviving for a certain number of years after a patient has already survived for a specified period [13]. For example, the 3‐year CS probability for a 1‐year OS indicates the likelihood of surviving an additional 3 years after already surviving for 1 year (i.e., surviving until Year 4). Building upon the concept of CS, conditional LRRFS, and DMFS are defined as the probability of continuing to survive for a specific number of years assuming that a patient does not experience local, regional, or remote recurrence after surviving for a certain period of time.

We assessed the annual recurrence probabilities of local–regional and distant recurrence in LARC patients. If the annual recurrence risk was below 5%, the follow‐up frequency would be scheduled annually. For annual risks between 5% and 15%, follow‐ups would be arranged every 6 months. If the annual recurrence risk was above 15%, follow‐ups were planned every 3 months.

### 2.6 Statistical Analyses

All statistical analyses were conducted using R software (version 4.2.2) and IBM SPSS Statistics software (version 26). The optimal cutoff values for BMI, PNI, and tumor size were calculated based on receiver operating characteristic (ROC) curve analysis [14, 15, 16]. Logistic regression analysis evaluated whether clinicopathological factors and imaging factors were potential high‐risk recurrence factors. In univariate analysis, variables with *p* values < 0.05 were included in multivariate analysis to determine independent recurrence risk factors. The nomogram model was validated using calibration curves. Decision curve analysis (DCA) assessed the clinical utility of the nomogram. Diagnostic ROC curve was used to evaluate the specificity and sensitivity of model. Kaplan–Meier curves were used to analyze the CS rates and cumulative recurrence risks of the study population. All analyses were two‐tailed, and *p* values < 0.05 were considered statistically significant.

## 3 Results

### 3.1 Clinical Characteristics

We ultimately included 561 LARC patients, with baseline clinical characteristics as shown in Table 1. The median follow‐up time was 44.6 months.

**TABLE 1:** Baseline information of clinical characteristics, nutritional indicators, and imaging indicators of LARC.

Characteristic	No. of patients
Age (years)	
< 60	285 (50.8%)
≥ 60	276 (49.2%)
Gender	
Male	342 (61.0%)
Female	219 (39.0%)
TNM stage	
II	327 (58.3%)
III	234 (41.7%)
BMI	
< 20.28	110 (19.6%)
≥ 20.28	451 (80.4%)
PNI	
< 52.5	414 (73.8%)
≥ 52.5	147 (26.2%)
CEA	
< 5	210 (37.4%)
≥ 5	351 (62.6%)
MRF	
No	413 (73.6%)
Yes	148 (26.4%)
EMVI	
No	477 (85.0%)
Yes	84 (15.0%)
Vascular tumor thrombus	
No	303 (54.0%)
Yes	258 (46.0%)
Perineural invasion	
No	389 (69.3%)
Yes	172 (30.7%)
Tumor deposit	
No	480 (85.6%)
Yes	81 (14.4%)
Tumor size (cm)	
< 4.8	359 (64.0%)
≥ 4.8	202 (36.0%)

Abbreviations: BMI, body mass index; EMVI, extramural vascular invasion; MRF, mesorectal fascia invasion; PNI, prognostic nutritional index.

### 3.2 Logistic Regression to Determine Independent Recurrence Factors

As shown in Table 2, univariate logistic regression analyses indicated that gender, BMI, PNI, CEA, MRF, EMVI, vascular tumor thrombus, perineural invasion, tumor deposits, and tumor size were potential recurrence factors. Multivariate analyses showed that PNI (odds ratio [OR], 0.558; 95% confidence interval [CI] [0.335–0.931]; *p* = 0.025), EMVI (OR, 2.685; 95% CI [1.410–5.114]; *p* = 0.003), vascular tumor thrombus (OR, 2.067; 95% CI [1.318–3.243]; *p* = 0.002), perineural invasion (OR, 3.200; 95% CI [2.038–5.024]; *p* < 0.001), and tumor size (OR, 2.749; 95% CI [1.782–4.240]; *p* < 0.001) were independent risk factors.

**TABLE 2:** Univariate and multivariate logistic regression analysis of recurrent factors in LARC.

Variables	Univariate analysis		Multivariate analysis	
	HR (95% CI)	*p* value	HR (95% CI)	*p* value
Age (years)				
≥ 60 vs. < 60	0.849 (0.589–1.224)	0.381		
Gender				
Male vs. female	0.657 (0.447–0.965)	0.032	0.819 (0.529–1.267)	0.369
TNM stage				
II vs. III	0.950 (0.657–1.375)	0.787		
BMI				
≥ 20.28 vs. < 20.28	0.617 (0.398–0.957)	0.031	0.641 (0.387–1.062)	0.084
PNI				
≥ 52.5 vs. < 52.5	0.608 (0.390–0.947)	0.028	0.558 (0.335–0.931)	0.025
CEA				
< 5 vs. ≥ 5	0.615 (0.424–0.892)	0.010	0.879 (0.572–1.351)	0.557
MRF				
Yes vs. no	1.837 (1.234–2.736)	< 0.001	1.152 (0.666–1.992)	0.613
EMVI				
Yes vs. no	2.75 (1.850–4.784)	< 0.001	2.685 (1.410–5.114)	0.003
Vascular tumor thrombus				
Yes vs. no	3.320 (2.261–4.876)	< 0.001	2.067 (1.318–3.243)	0.002
Perineural invasion				
Yes vs. no	4.459 (3.014–6.598)	< 0.001	3.200 (2.038–5.024)	< 0.001
Tumor deposit				
Yes vs. no	2.388 (1.474–3.869)	< 0.001	1.732 (0.983–3.049)	0.057
Tumor size (cm)				
≥ 4.8 vs. < 4.8	2.472 (1.698–3.598)	< 0.001	2.749 (1.782–4.240)	< 0.001

Abbreviations: BMI, body mass index; EMVI, extramural vascular invasion; MRF, mesorectal fascia invasion; PNI, prognostic nutritional index.

### 3.3 Constructing Nomogram and Risk Stratification

**FIGURE 1:**
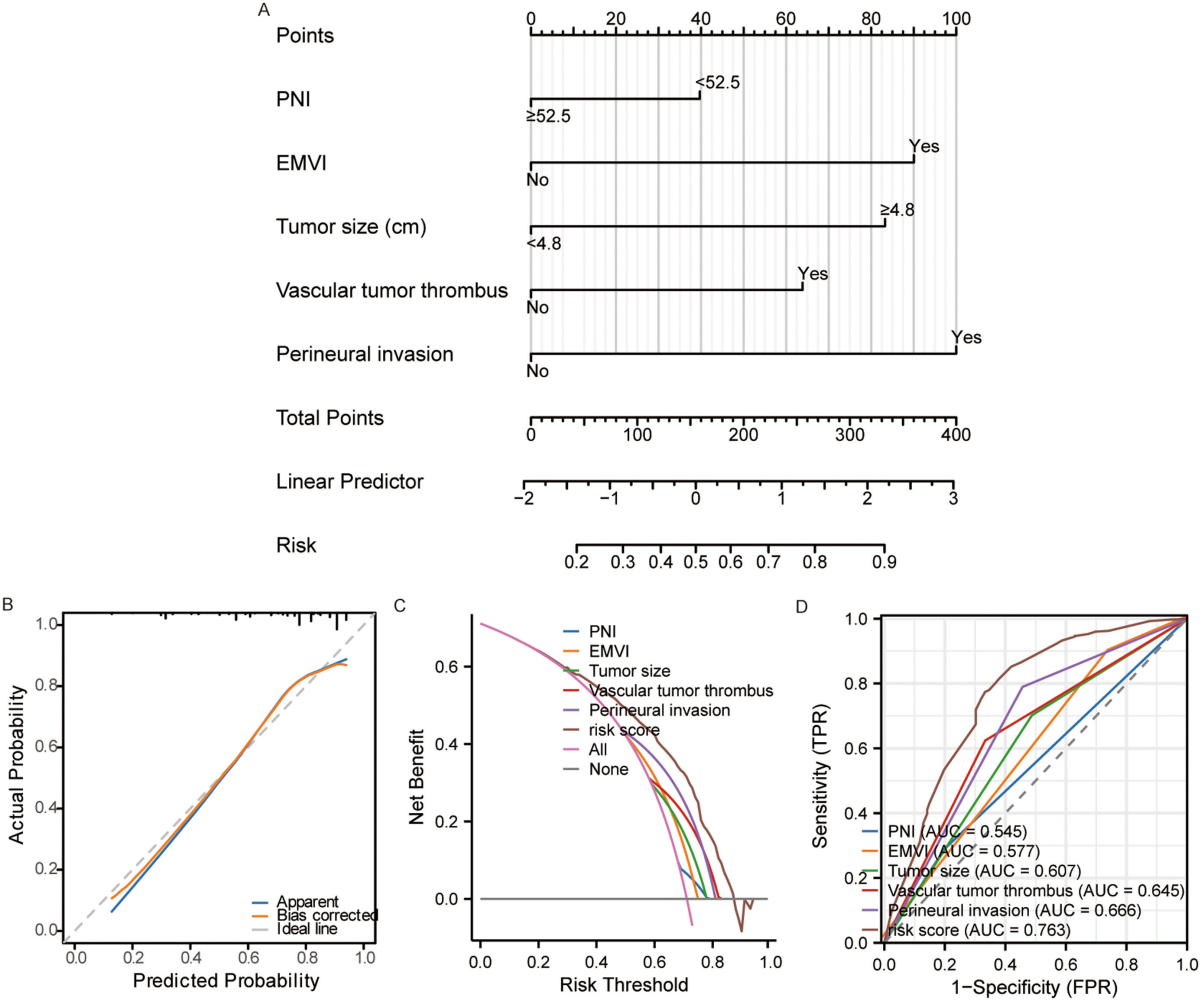
(A) Nomogram was constructed by independent recurrence factors, including PNI, EMVI, vascular tumor thrombus, perineural invasion, and tumor size. (B) The calibration curve suggested good consistency between predicted and actual recurrence probabilities. (C) DCA indicated that the risk model provides greater clinical benefit than each individual recurrent factor. (D) The model's AUC value of 0.763 indicated high specificity and sensitivity than each individual recurrent factor. DCA, decision curve analysis; EMVI, extramural vascular invasion; PNI, prognostic nutritional index.

Based on the aforementioned independent risk factors, we constructed a nomogram model (Figure 1A). The performance of the nomogram model was assessed using calibration curves and decision curves. Calibration curve results demonstrated good consistency between the actual observed outcomes and predicted probabilities (Figure 1B). DCA indicated that the model provided greater clinical benefit compared with individual risk factors alone (Figure 1C). An area under the curve (AUC) of 0.763 suggested that the model exhibits good sensitivity and specificity (Figure 1D).

Subsequently, the total risk score is the score of each independent sample in each indicator of the model calculated according to the nomogram model, and the total risk score of each independent sample is obtained after summing up. The median calculated overall risk score was 127 (range, 0–377). Then the total risk score was divided into three groups: low‐risk group (≤ 125), moderate‐risk group (126 < score ≤ 252), and high‐risk group (252 < score ≤ 377). Survival curves showed statistically significant differences in OS, PFS, LRRFS, and DMFS among patients in different risk groups (*p* < 0.05) (Figure S1A–D).

### 3.4 Conditional Survival and Recurrence Risk

#### 3.4.1 Different Risk Groups

**FIGURE 2:**
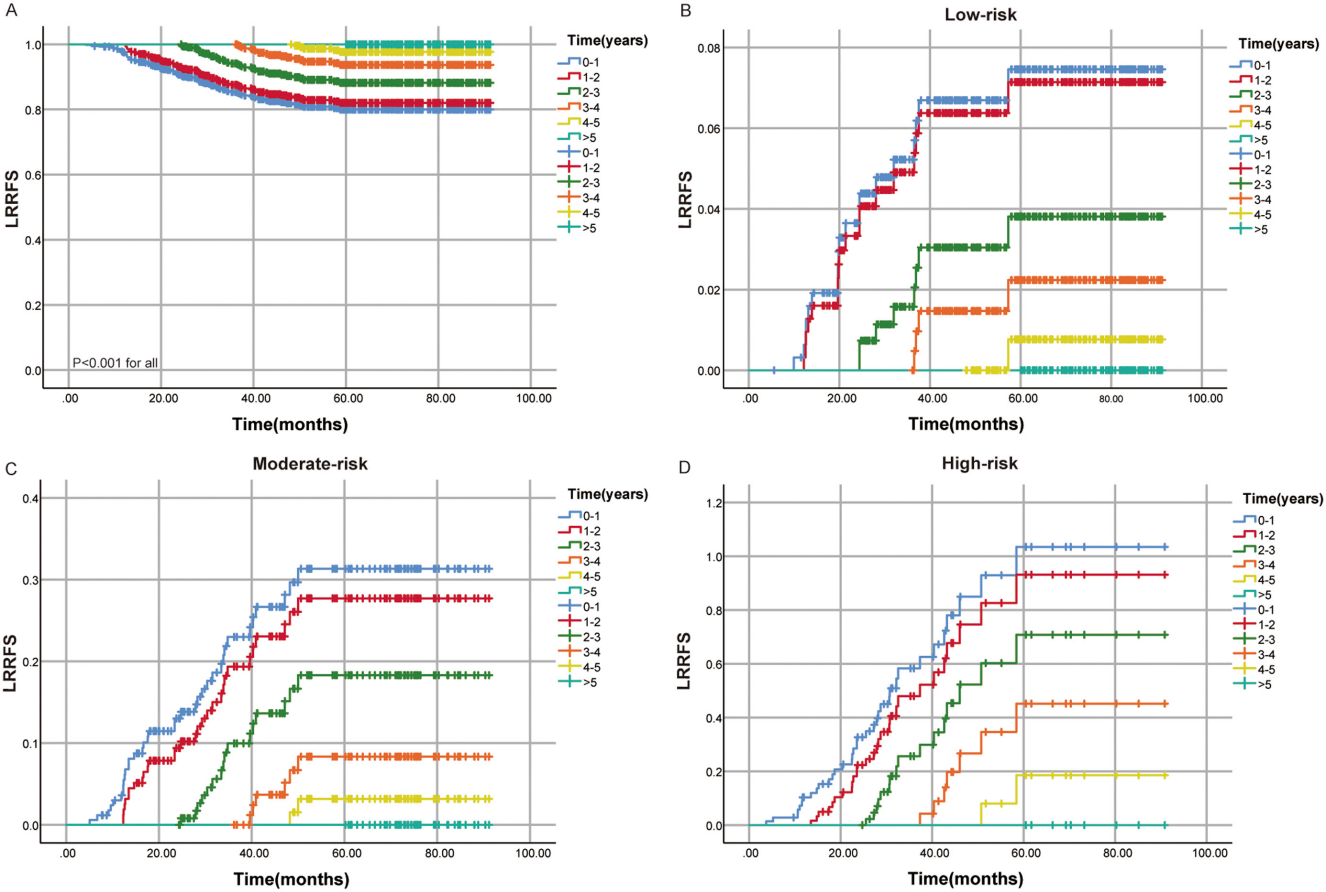
(A) Conditional survival probabilities of LRRFS in different risk groups. (B) Cumulative recurrence rate of LRRFS in the low‐risk group. (C) Cumulative recurrence rate of LRRFS in the moderate‐risk group. (D) Cumulative recurrence rate of LRRFS in the high‐risk group. LRRFS, local‐regional recurrence‐free survival.

As shown in Figure 2A, the conditional LRRFS survival probability increased over the years. The 5‐year LRRFS is 80.0%. For patients surviving 1, 2, and 3 years, their 5‐year conditional LRRFS were 82.0%, 88.2%, and 93.7%, respectively. There is a significant increase in 5‐year conditional LRRFS in the middle 3 years after treatment, while the increase is relatively small in the first and fifth years after treatment. Across different risk groups, as the posttreatment survival time extends, the risk of local recurrence decreased annually (Figure 2B–D).

**FIGURE 3:**
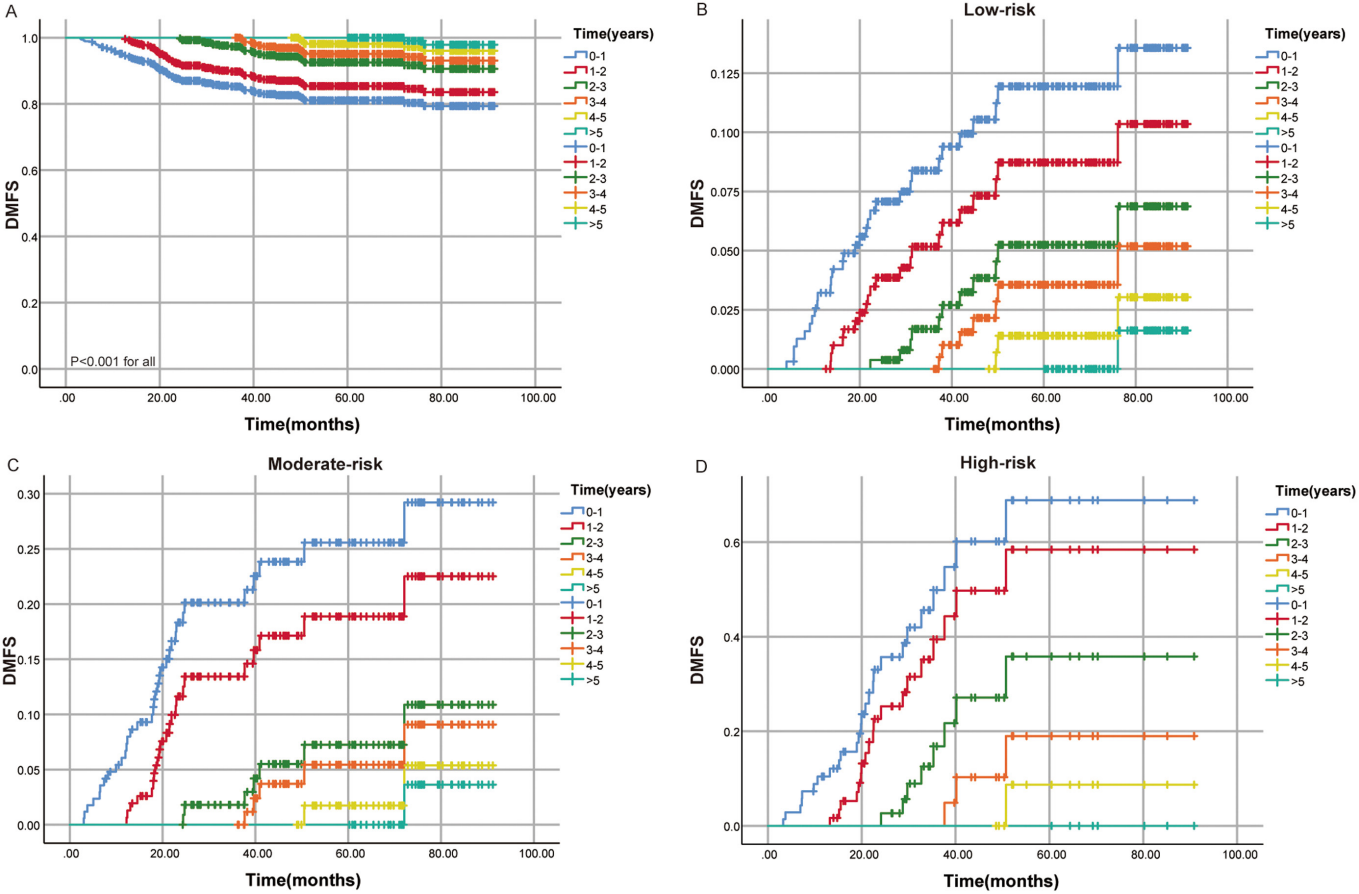
(A) Conditional survival probabilities of DMFS in different risk groups. (B) Cumulative recurrence rate of DMFS in the low‐risk group. (C) Cumulative recurrence rate of DMFS in the moderate‐risk group. (D) Cumulative recurrence rate of DMFS in the high‐risk group. DMFS, distant metastasis‐free survival.

As depicted in Figure 3A, conditional DMFS significantly improved with prolonged postcomprehensive treatment survival time, with a 5‐year DMFS of 81.1% (Figure 3A). For patients with a 1‐year survival, the 5‐year conditional DMFS increased to 85.4%, for those with a 2‐year survival, it increased to 92.5%, and for those with a 3‐year survival, it reached 95.1%. The 4‐year survival rate is 98.1%. The 5‐year conditional DMFS showed a substantial increase in the first 3 years after comprehensive treatment, followed by a decrease in the rates of increase in the fourth and fifth years. Similarly, across different risk groups, the risk of distant recurrence decreases year by year as the posttreatment survival time extends (Figure 3B–D).

#### 3.4.2 Different Clinical Stages

**FIGURE 4:**
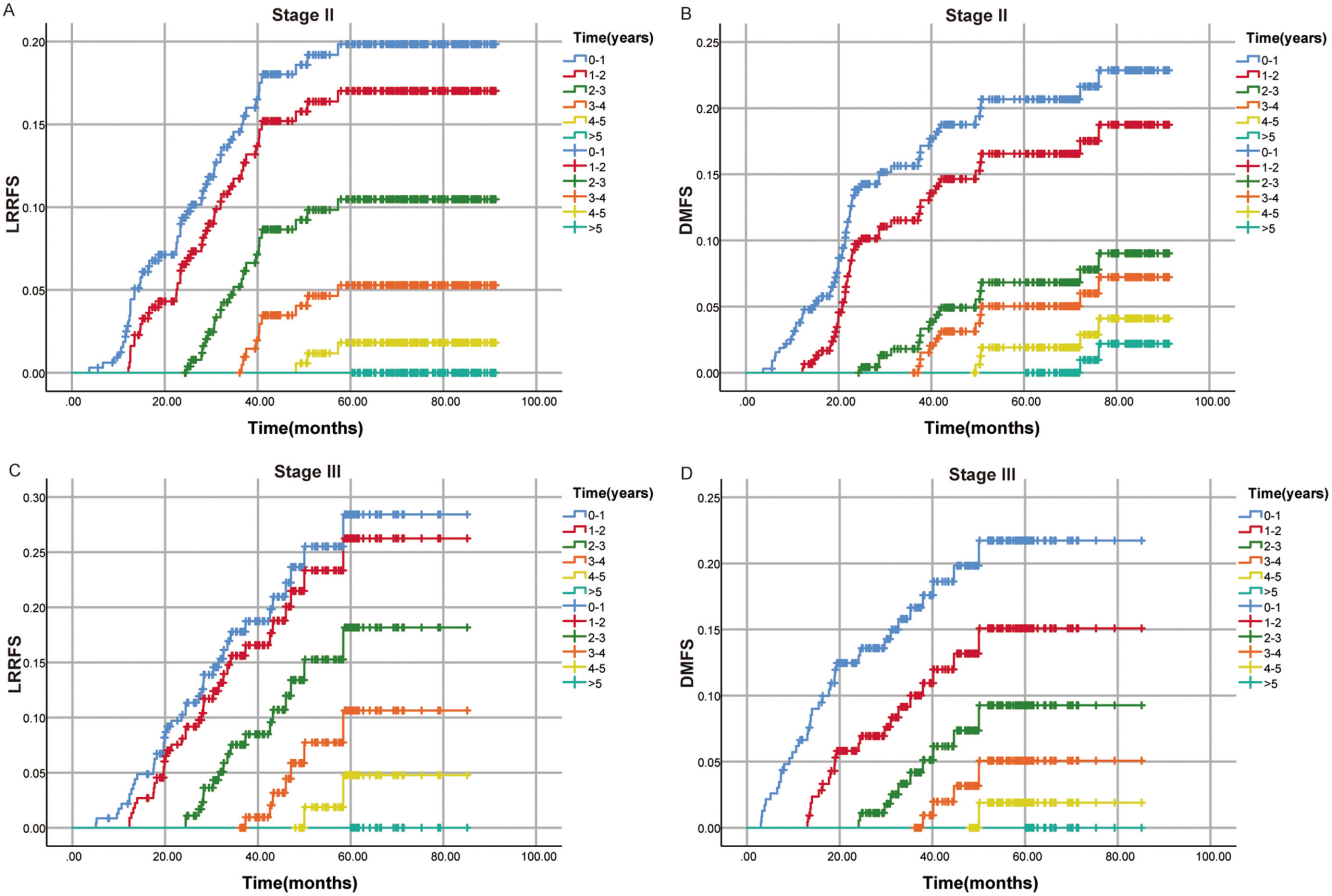
(A) Cumulative recurrence rate of LRRFS in the Stage II. (B) Cumulative recurrence rate of DMFS in the Stage II. (C) Cumulative recurrence rate of LRRFS in the Stage III. (D) Cumulative recurrence rate of DMFS in the Stage III. DMFS, distant metastasis‐free survival; LRRFS, local‐regional recurrence‐free survival.

Figure 4 displayed the recurrence risks of local–regional and distant recurrence in RC patients after comprehensive therapy for Stage II and Stage III. We observed that posttreatment, both Stage II and Stage III patients experienced a yearly decrease in their rates of local–regional and distant recurrence as their survival time extends (Figure 4A–D).

### 3.5 Annual Recurrence Risk and Optimal Follow‐Up Strategies

#### 3.5.1 Different Risk Groups

**FIGURE 5:**
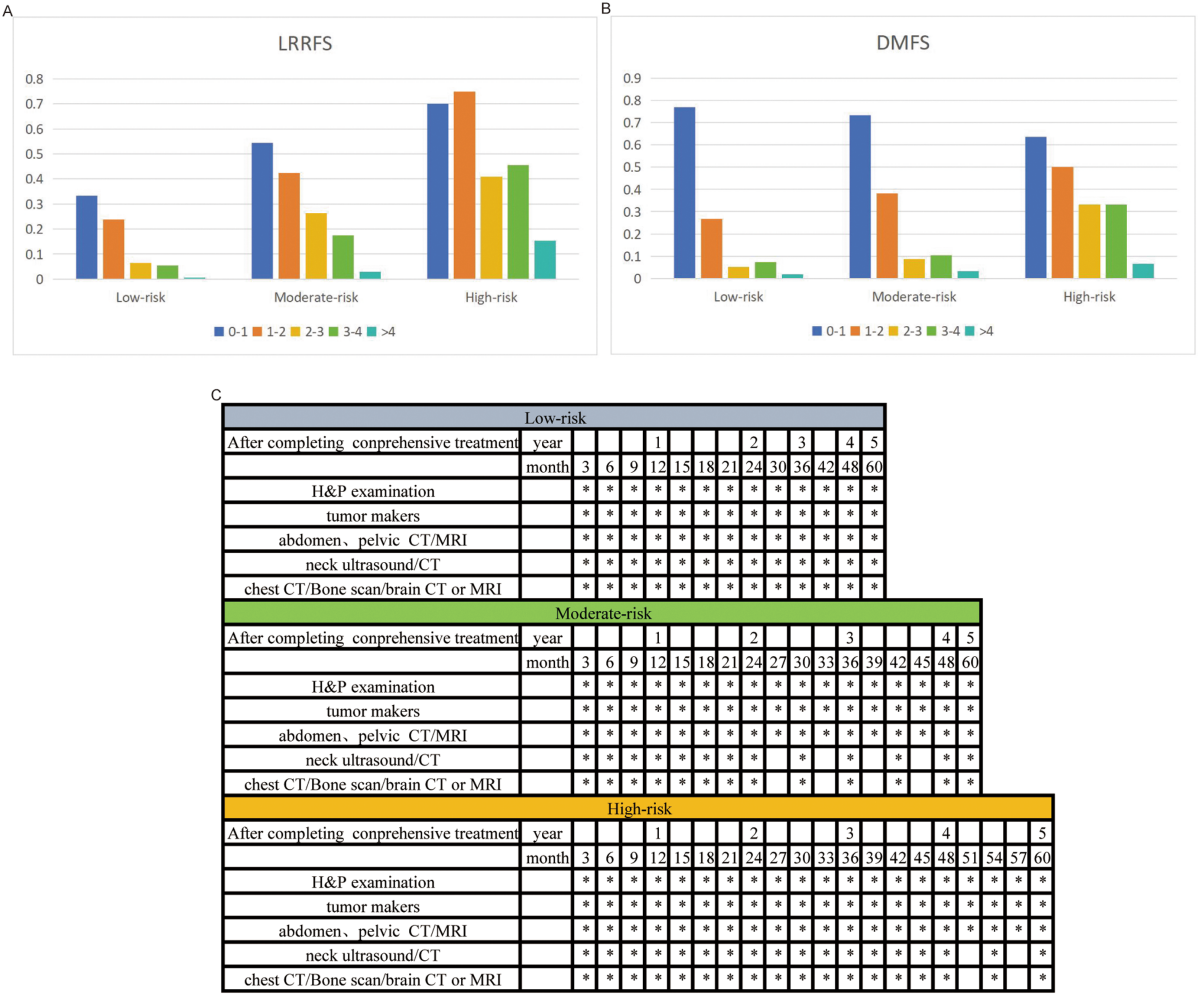
(A) Table of cumulative recurrence risk probability for LRRFS in different risk groups. (B) Table of cumulative recurrence risk probability for DMFS in different risk groups. (C) Individualized follow‐up strategies based on different risk groups. DMFS, distant metastasis‐free survival; LRRFS, local‐regional recurrence‐free survival.

The detailed information on annual local–regional and distant recurrence risks for different risk groups was illustrated in Figure 5A,B. For the low‐risk group, the local–regional and distant annual recurrence rates exceeded 15% in the first and second years, while falling between 5% and 15% in the third and fourth years. By the fifth year, the rate dropped below 5%. The intermediate‐risk group exhibited an annual local–regional recurrence risk exceeding 15% for the first 4 years and dropping below 5% in the fifth year, while the distant recurrence risk was above 15% for the first 2 years, between 5% and 15% in the third and fourth year, and below 5% in the fifth year. The high‐risk group consistently experienced an annual local–regional recurrence risk exceeding 15%, while the distant recurrence risk was above 15% for the first 4 years and dropped between 5% and 15% in the fifth year. In summary, the local and distant recurrence risks in the moderate‐ and high‐risk groups were notably higher than those in the low‐risk group, indicating that patient risk of recurrence increased with the risk level.

Based on our predetermined criteria, we have developed a posttreatment follow‐up model for LARC patients in different risk groups based on the cumulative risk of treatment failure each year. As shown in Figure 5C, all patients undergo a comprehensive assessment in the first 3 months following comprehensive treatment to establish baseline standards for subsequent follow‐ups. Due to the low recurrence risk in the low‐risk group, imaging examinations were recommended every 3 months during the first and second years, as the local–regional and distant recurrence rates exceeded 15%. In the third and fourth years, with recurrence risks between 5% and 15%, follow‐up visits were advised every 6 months. For the fifth year, if the annual recurrence rate was below 5%, annual follow‐up visits were recommended. For the moderate‐risk group, with recurrence risks exceeding 15% in the first 2 years, follow‐up visits were scheduled every 3 months during this period. In the third and fourth year, as the risk of local recurrence exceeds 15%, follow‐ups every 3 months were advised, while the risk of distant recurrence falls between 5% and 15%, suggesting follow‐ups every 6 months. Subsequently, yearly follow‐ups were recommended. The high‐risk group, with the highest risk of recurrence, was advised to have follow‐up visits every 3 months for the first 4 years. In the fifth year, monitoring for local‐regional recurrence is recommended every 3 months, with monitoring for distant recurrence within 6 months.

#### 3.5.2 Different Clinical Stages

**FIGURE 6:**
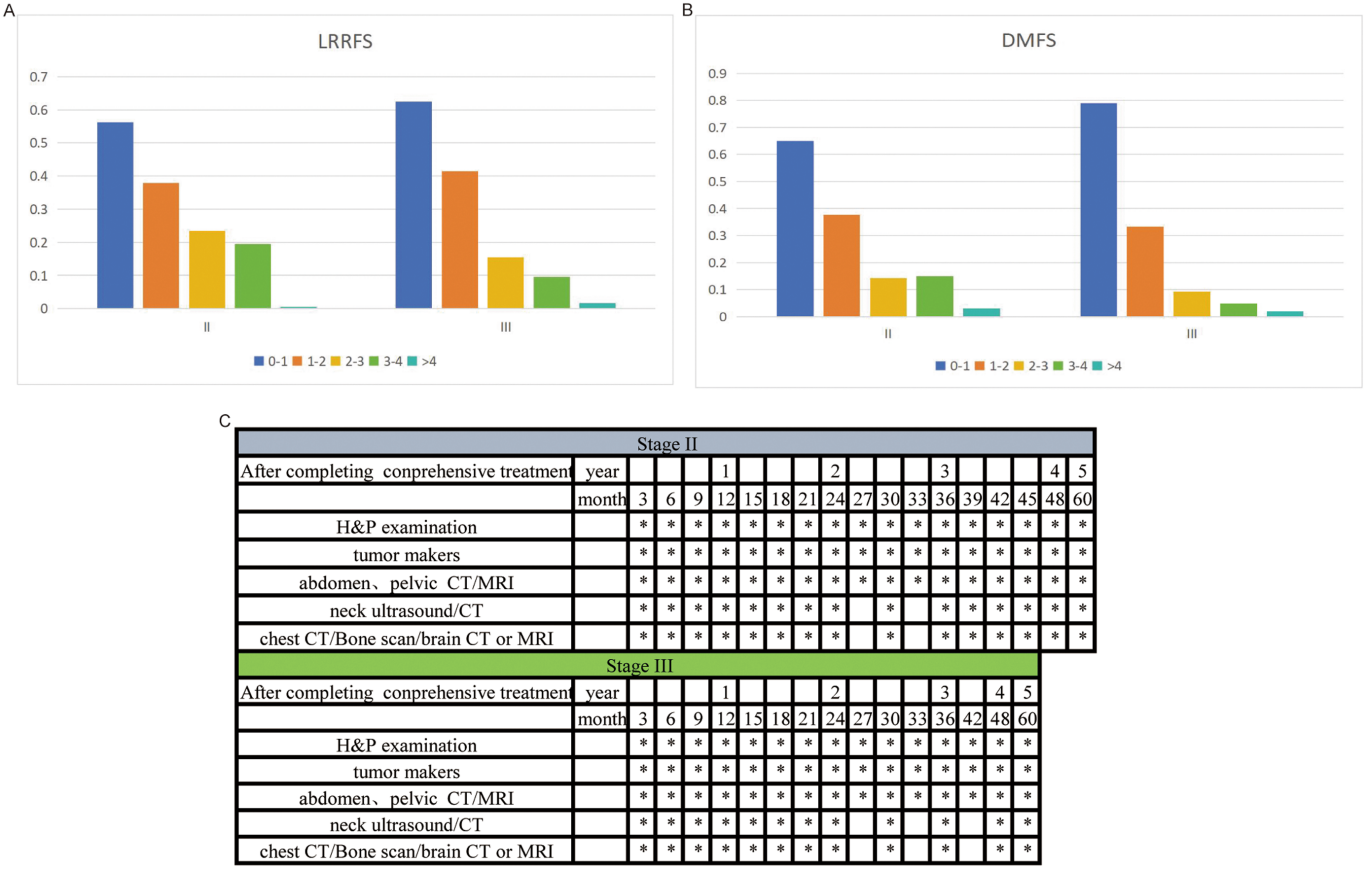
(A) Table of cumulative recurrence risk probability for LRRFS in clinical stages. (B) Table of cumulative recurrence risk probability for DMFS in clinical stages. (C) Individualized follow‐up strategies based on clinical stages. DMFS, distant metastasis‐free survival; LRRFS, local‐regional recurrence‐free survival.

The annual local–regional and distant recurrence risks for Stages II and III patients were shown in Figure 6A,B, with follow‐up strategies outlined in Figure 6C. For Stage II patients, the risk of recurrence was higher in the first 4 years, with both local–regional and distant recurrence rates exceeding 15%. Therefore, it was recommended to have follow‐up visits every 3 months for the first 4 years. With the recurrence risk dropping below 5% in the fifth year, yearly follow‐ups were advised. For Stage III patients, the risk of local recurrence exceeds 15% in the first 3 years, warranting follow‐up visits every 3 months during this period. In the fourth year, with the recurrence risk ranging from 5% to 15%, follow‐up visits every 6 months were recommended, and with a risk below 5% in the fifth year, yearly follow‐ups were advised. Regarding distant recurrence for Stage III patients, the risk exceeded 15% in the first 2 years, suggesting follow‐up visits every 3 months. In the third year, with the risk falling between 5% and 15%, follow‐up visits every 6 months were advised, and with risks below 5% in the fourth and fifth years, yearly follow‐ups were recommended. During the follow‐up period, clinicians can adjust the follow‐up strategy appropriately according to the symptoms and imaging findings of patients.

## 4 Discussion

LARC refers to the situation where cancer cells invade the local deep tissues of the rectal wall and nearby lymph nodes, but distant metastasis has not occurred yet. However, even after receiving comprehensive treatment, many patients still experience recurrence after the completion of treatment. The prognosis of recurrent LARC is often poor, and treatment is more challenging. Close follow‐up after comprehensive treatment is crucial for the early detection of recurrence or metastasis, and timely intervention to improve patient prognosis and quality of life. Unfortunately, there is currently a lack of research on individualized follow‐up strategies. Although clinical guidelines provide follow‐up recommendations for LARC patients, most are based on expert opinions or clinical practice. Therefore, developing more personalized follow‐up strategies based on risk stratification for different groups of patients has become an urgent issue. This study identified high‐risk recurrence factors for LARC, stratified the risks, and introduced CS to dynamically assess changes in recurrence risk. Through the development of this study, it is expected to provide more scientific and personalized guidance for the treatment and follow‐up of patients with LARC.

To the best of my knowledge, this study is the first to assess the CS and dynamic recurrence risk in LARC patients after comprehensive treatment. Our results indicated that PNI, EMVI, vascular tumor thrombus, perineural invasion, and tumor size are independent factors for recurrence in LARC, and we constructed a nomogram model. Calibration curves, DCA, and AUC suggested that the model has good clinical applicability and can aid clinicians in their decision‐making. Kaplan–Meier curves show significantly better survival in the low‐risk group compared to the moderate‐high‐risk group. Conditional LRRFS and DMFS curves indicated that as survival time increases, the patient's survival probability also increases. The cumulative recurrence risk for different risk groups decreases annually as the years' progress.

Research indicated that nutritional indicators have a significant impact on the recurrence and prognosis of LARC patients [17, 18, 19]. PNI, a comprehensive indicator considering immune function and protein levels in the body, is correlated with higher recurrence rates and poorer prognosis when lower [20]. A study highlighted that LARC patients with a PNI below 45 had significantly reduced survival rates [21]. These findings align with our research results. Another study found that decreased serum protein and lymphocyte count were associated with increased risk of recurrence and poor prognosis in cancer patients [22]. Therefore, a comprehensive assessment of PNI, as a nutritional marker, is crucial for evaluating the recurrence risk and prognosis of LARC patients, aiding in the development of personalized treatment plans and enhancing long‐term survival rates.

In patients with LARC, several imaging related indexes play crucial roles in influencing the recurrence and prognosis of the disease. Among these factors, EMVI, vascular tumor thrombus, perineural invasion, and tumor size have been identified as key contributors to the aggressive behavior of the tumor and its potential impact on patient outcomes. EMVI refers to the invasion of tumor cells into the veins located outside the muscularis propria of the rectal wall. Studies have shown that the presence of EMVI is associated with a higher risk of local recurrence, distant metastasis, and poorer overall survival in LARC patients [23, 24]. EMVI serves as an indicator of tumor aggressiveness and is considered a negative prognostic factor in predicting disease progression [25]. Vascular tumor thrombus occurs when tumor cells invade the blood vessels, leading to the formation of thrombi within the vessels. The presence of vascular tumor thrombus is associated with a higher risk of tumor dissemination and metastasis, which can significantly impact the recurrence rate and prognosis of LARC patients [26]. The presence of vascular tumor thrombus indicates a more advanced stage of the disease and is linked to poorer outcomes in terms of survival and disease‐free intervals. Perineural invasion refers to the infiltration of tumor cells into the nerve sheaths surrounding the nerves in the perirectal area [27]. Perineural invasion has been linked to an increased risk of local recurrence, distant metastasis, and decreased overall survival in LARC patients. Tumors exhibiting perineural invasion are often associated with a more aggressive phenotype and are less responsive to treatment, leading to a higher likelihood of disease recurrence [28]. We also found that tumor size is a significant factor in determining the recurrence of LARC patients. Larger tumor size is often associated with a higher risk of local recurrence, lymph node involvement, and distant metastasis [29]. Tumor size reflects the extent of tumor burden and aggressiveness, influencing treatment outcomes and patient survival. In conclusion, factors such as EMVI, vascular tumor thrombus, perineural invasion, and tumor size play crucial roles in shaping the clinical course, recurrence risk, and prognosis of LARC. Understanding the impact of these pathologic factors on disease progression can aid in risk stratification, treatment decision‐making, and the development of personalized therapeutic strategies to improve outcomes for LARC patients.

In patients with LARC who have undergone curative surgery and adjuvant treatment, approximately 30% experience recurrence, with about 90% of recurrences occurring within the first 5 years post comprehensive treatment, the majority of which are detected within the first 3 years [30, 31, 32]. Currently, there is no consensus among major societies regarding the optimal follow‐up strategy for these patients. Previous meta‐analyses comparing low‐density and high‐density monitoring regimens have demonstrated the advantage of more intensive follow‐up in Stage II or III patients post‐treatment [33, 34, 35]. However, these studies have overlooked individual variations in patients and patterns of treatment failure.

To address this challenge, we conducted a comprehensive analysis of the evolution of patterns of local–regional and distant treatment failures in different risk groups and clinical stages, providing more specific guidance for follow‐up strategies. Our dynamic analysis based on CS results indicated that patients in the intermediate to high‐risk groups have significantly higher recurrence risks compared with the low‐risk group. In response to these risks, we have developed a posttreatment follow‐up model for LARC patients in different risk groups. Low‐risk group patients require close monitoring in the first 2 years, whereas those in the intermediate to high‐risk groups necessitate intensive follow‐up for the first 4 years. For Stage II patients, it is recommended to have follow‐up visits every 3 months for the first 4 years, followed by annual visits thereafter. Stage III patients are advised to have follow‐up visits every 3 months for the first 3 years, every 6 months in the fourth year, and then annually. The follow‐up frequency may be adjusted based on symptoms and imaging findings during the follow‐up process. The stage‐based follow‐up strategy indicated that Stage III patients were followed less frequently than Stage II patients during the fourth year of surveillance. Since we are based on available clinical data, it may fail to adequately account for other important factors that influence the frequency of follow‐up, such as overall patient health, treatment response, and other clinical characteristics. This may have resulted in less frequent follow‐up of Stage III patients than expected in some cases. In addition, tumor staging is an important indicator to evaluate the prognosis of patients, but relying on staging alone may not fully reflect the individual differences and disease progression of patients. Therefore, it is recommended that other clinical indicators and patient characteristics should be taken into account when formulating follow‐up plans to ensure a more personalized and effective follow‐up strategy.

The follow‐up strategies based on different risk groups offer a more personalized and tailored approach compared with strategies based solely on clinical staging. By considering individual patient variations and patterns of treatment failure, the risk‐based follow‐up strategies provide a more precise estimation of recurrence risks and appropriate follow‐up intensity. This allows for early detection and intervention in high‐risk patients while minimizing unnecessary visits for low‐risk patients and optimizing resource allocation. In contrast, clinical staging‐based follow‐up strategies may overlook the nuances and variability in recurrence risks within each stage. They may lead to either overmonitoring low‐risk patients, causing undue anxiety and resource burden, or undermonitoring high‐risk patients, potentially missing early signs of recurrence. Risk‐based follow‐up strategies, by focusing on the specific risk profile of each patient, enable a more efficient use of healthcare resources, enhance patient outcomes, and improve overall quality of care.

This study is the first to develop a follow‐up strategy for LARC patients based on high‐risk recurrence factors and CS, but it has certain limitations. Firstly, it is a retrospective single‐center study, inevitably leading to some selection bias. Secondly, the study did not comprehensively consider all relevant treatment factors, such as chemotherapy regimens, inflammatory markers, and pathological factors, which may influence the recurrence risk and patient prognosis in LARC. Thirdly, the study only focused on LARC and may not have generalizability to patients with other clinical stages. Fourth, although we developed these thresholds on the basis of clinical experience and existing clinical practice guidelines, systematic studies to validate these choices are lacking. Therefore, future studies should focus on exploring and verifying the optimal follow‐up frequency under different annual recurrence risk levels to provide a more solid theoretical basis. Finally, the lack of external validation in this study highlights the need for multicenter studies to further confirm the study results. Addressing these limitations in future studies would contribute to a more thorough understanding of personalized follow‐up strategies for LARC patients and improve the precision and effectiveness of clinical management in this patient population.

## 5 Conclusion

In conclusion, the follow‐up strategy for patients with LARC should be adjusted based on individual risk characteristics rather than solely relying on clinical staging. By incorporating risk factors such as PNI, EMVI, vascular tumor thrombus, perineural invasion, and tumor size, and utilizing dynamic assessment of recurrence risk through CS, personalized follow‐up strategies have been developed to provide a more intricate and targeted approach to monitoring recurrence and metastasis. Tailoring follow‐up strategies to individual risk profiles is crucial for optimizing healthcare resources, enhancing treatment outcomes, and improving the long‐term prognosis of patients with LARC.

## Author Contributions

**Jianjian Qiu:** conceptualization (equal), formal analysis (equal), methodology (equal), writing – original draft (equal), writing – review and editing (equal). **Yilin Yu:** conceptualization (equal), data curation (equal), formal analysis (equal), methodology (equal), writing – original draft (equal), writing – review and editing (equal). **Zhiping Wang:** conceptualization (equal), formal analysis (equal), methodology (equal), writing – original draft (equal), writing – review and editing (equal). **Liang Hong:** data curation (equal), formal analysis (equal), methodology (equal), supervision (equal). **Lingdong Shao:** data curation (equal), formal analysis (equal), methodology (equal), supervision (equal). **Junxin Wu:** funding acquisition (equal), supervision (equal).

## Ethics Statement

This study was sanctioned by the Fujian Cancer Hospital's Ethics Committee. All patients provided written informed consent.

## Consent

The manuscript has been approved by all authors for publication.

## Conflicts of Interest

The authors declare no conflicts of interest.

## Supporting information


Figure S1.

## Data Availability

The data supporting the results of this study can be provided by the corresponding author upon reasonable request.
