# Supplementary figures and images for: Developing Individualized Follow‐Up Strategies Based on High‐Risk Recurrence Factors and Dynamic Risk Assessment for Locally Advanced Rectal Cancer
Source: Cancer Med. 2024 Oct 28;13(20):e70323. doi: 10.1002/cam4.70323 (PMC11516045; doi:10.1002/cam4.70323)

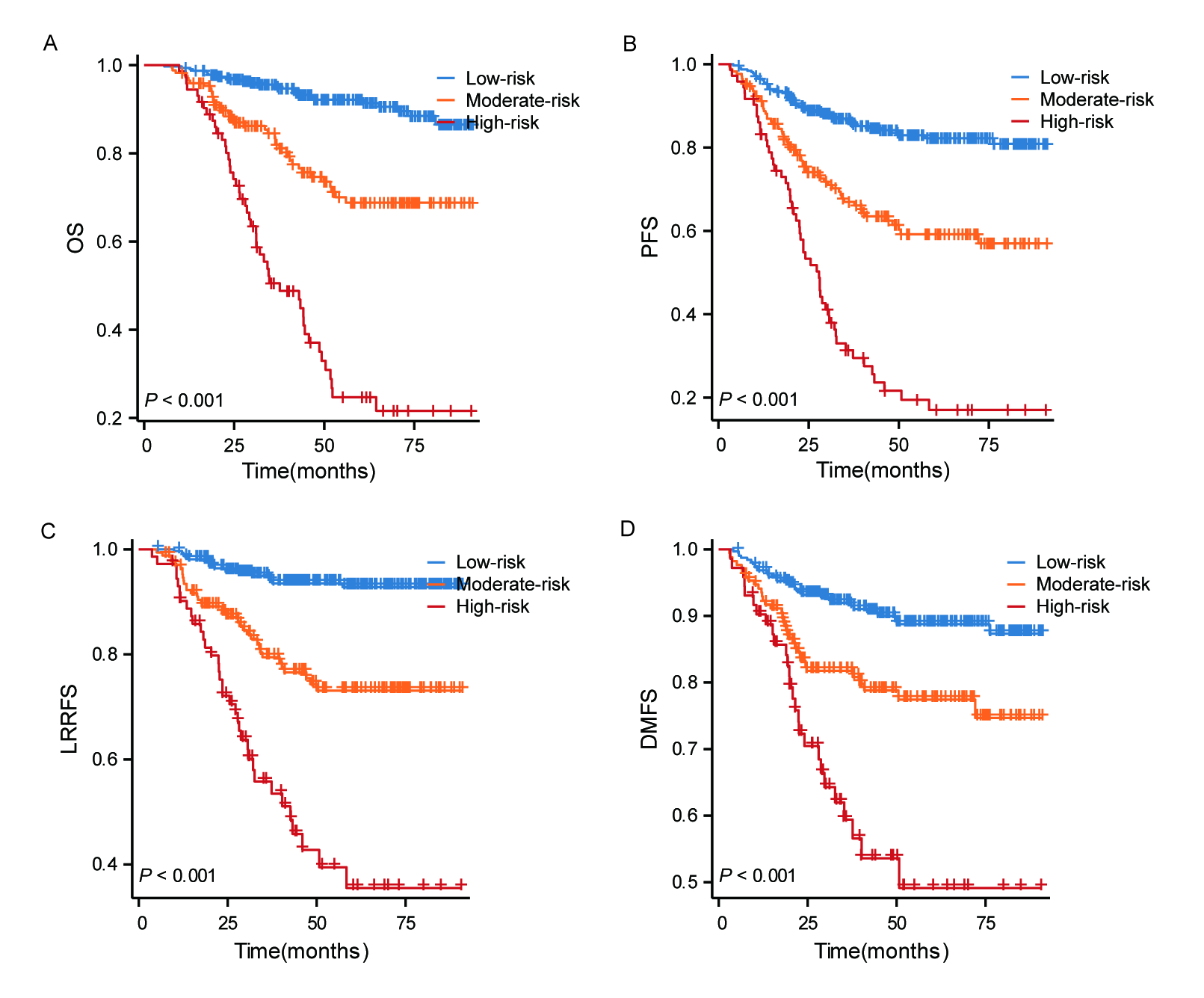

Supplement: Supplementary file 1 — Figure S1. [file CAM4-13-e70323-s001.tif]
